# Regional disparities and sociodemographic determinants of food insecurity in Somalia: a secondary cross-sectional analysis of a National survey

**DOI:** 10.1186/s41043-025-01078-9

**Published:** 2025-10-08

**Authors:** Yusuf Hared Abdi, Yakub Burhan Abdullahi, Mohamed Sharif Abdi, Sharmake Gaiye Bashir, Naima Ibrahim Ahmed, Ahmed Abdiaziz Alasow, Gallad Dahir Hassan

**Affiliations:** 1 Department of Environmental Science, Faculty of Geoscience & Environment, Hormuud University, Mogadishu, Somalia; 2 Demartino Public Hospital, Mogadishu, Somalia; 3 Department of Environmental Science and Technology, Faculty of Forestry and Environment, University Putra Malaysia, 43400 Serdang, Selangor Malaysia; 4 School of Public Health, College of Health Sciences, Makerere University, Kampala, Uganda

## Abstract

**Background:**

Food insecurity represents a critical public health challenge in Somalia, where four decades of state fragility, environmental vulnerability, and sociopolitical instability have created chronic food access limitations. Although existing research has primarily examined macro-level determinants, there remains a substantial gap in understanding how individual-level sociodemographic factors influence food security outcomes among various population subgroups.

**Methods:**

This cross-sectional study analyzed data from the Somalia Demographic and Health Survey involving 52,154 participants aged 13 years and above. A multistage stratified cluster sampling technique ensured representation across urban, rural, and nomadic populations. Bivariate and multivariate logistic regression analyses were used to examine the associations between sociodemographic characteristics and household food insecurity, with adjusted odds ratios calculated to identify independent predictors.

**Results:**

Significant disparities were observed across multiple dimensions. Educational attainment demonstrated strong protective effects, with higher education reducing food insecurity odds by 82% compared with no formal education. Middle-aged adults divorced and widowed individuals, and female-headed households showed elevated vulnerability. Extreme regional heterogeneity was observed, with residents in Bakool and Gedo facing more than 13 times greater risk than those in Awdal. Rural and nomadic populations had significantly higher odds than urban dwellers.

**Conclusion:**

Food insecurity in Somalia operates through complex, intersecting pathways of sociodemographic vulnerability and pronounced geographical inequalities. Food insecurity in Somalia results from a complex, multifaceted crisis spanning social, political, economic, and environmental domains. Effective interventions must simultaneously address educational infrastructure development, provide targeted support for vulnerable demographic groups, and implement place-based strategies that recognize the extreme geographic clustering of vulnerability across Somalia’s diverse regions.

## Introduction

Food insecurity represents one of the most pressing public health challenges of our time and is fundamentally defined as a deficit in households’ access to appropriate food in adequate quantity and quality due to limited financial resources or other constraining factors [1]. According to the Food and Agriculture Organization (FAO), food security exists when all people, at all times, have physical and economic access to sufficient, safe and nutritious food that meets their dietary needs and food preferences for an active and healthy life [2–4]. The global magnitude of this crisis is staggering, with 800 million individuals lacking access to sufficient food, over 2 billion people experiencing key micronutrient deficiencies, and 60% of individuals in low-income countries facing food insecurity [4]. According to FAO estimates, 29.6% of the global population, comprising 2.4 billion people, experienced moderate to severe food insecurity in 2022, with 11.3% suffering from severe food insecurity (FAO, 2023) [5]. The COVID-19 pandemic and geopolitical conflicts, particularly the war in Ukraine, have exacerbated these figures, with UN Secretary-General António Guterres noting that “global hunger levels are at a new high,” and experts predict that an additional 50 million people will be affected by hunger [6, 7]. This crisis extends beyond traditional assumptions about poverty-stricken regions, as studies demonstrate unexpectedly high food insecurity prevalence rates of 8 to 20%, even in high-income countries, challenging perceptions that this is solely a developing world issue [8]. Food insecurity is a critical social determinant of health, encompassing the social, economic, political, cultural, and environmental conditions under which people are born, grow, live, work, and age [9, 10]. The interconnected nature of food insecurity with broader social determinants creates what researchers describe as a problematic web of inequalities that negatively affects human physical, social, emotional, and cognitive development throughout the life course, while simultaneously serving as a major environmental disruptor with serious implications for planetary health [4, 8]. The urgency of addressing this global challenge is underscored by the recognition that current progress toward achieving Sustainable Development Goal 2, ending hunger by 2030, remains significantly off-track, with the proportion of the world’s population facing hunger showing little improvement since 2015 [6, 7, 11].

Sub-Saharan Africa remains the epicenter of global food insecurity challenges, with over 30% of its population experiencing severe food insecurity as of 2022, which is the highest regional prevalence worldwide [12, 13]. In particular, the Horn of Africa exemplifies a crisis nexus where climate shocks, political instability, and economic fragility intersect to create chronic food access challenges [14]. This region has witnessed a 45% increase in food-insecure populations since 2015, with Somalia consistently ranking among the most vulnerable nations owing to its exposure to recurrent droughts and protracted conflicts [15, 16]. The compounding effects of climate variability, characterized by erratic rainfall patterns and rising temperatures, have reduced agricultural productivity by an estimated 34% in semi-arid zones, exacerbating reliance on food imports and humanitarian aid [16, 17]. These regional dynamics create a complex landscape in which traditional coping mechanisms are increasingly insufficient against mounting environmental and sociopolitical pressures [14, 18].

Somalia’s food security landscape has been shaped by four decades of state fragility, with the collapse of central governance in 1991, triggering cyclical humanitarian crises that persist today [18, 19]. The country’s unique socio-political fabric comprising clan-based governance systems, widespread internal displacement (2.9 million internally displaced persons [IDPs] as of 2023), and active insurgency groups creates substantial barriers to equitable food distribution [20, 21]. Environmental vulnerability presents these challenges; 83% of Somalia’s territory is arid or semi-arid, with 70% of the population dependent on rain-fed agriculture and pastoralism [22]. Stark disparities emerge across geographies: urban centers like Mogadishu exhibit 22% lower food insecurity rates than rural agro-pastoralist communities, while IDP camps in Baidoa report acute malnutrition rates exceeding 30% [20, 22]. Gender disparities further stratify access, with female-headed households experiencing 1.5 times higher food insecurity due to limited asset ownership and cultural restrictions on mobility [21].

Existing researches on Somali food security disproportionately focuses on macro-level drivers such as climate change and conflict, neglecting the nuanced analysis of intersecting socio-demographic determinants [15]. While studies such as those by Kinda et al. (2019) and the FAO have established broad correlations between rainfall variability and food access, a critical gap remains in understanding individual-level factors, including educational attainment, marital status, and household composition, mediate vulnerability [17, 22]. National surveys frequently aggregate data at regional levels, obscuring disparities between nomadic pastoralists, the urban poor, and displaced populations [20, 21]. Furthermore, the 2018 Somalia Demographic and Health Survey (SDHS) revealed that 68% of food insecurity analyses rely on outdated poverty proxies, rather than multidimensional vulnerability indices [15]. This study addresses these gaps by employing disaggregated SDHS data to map how specific demographic variables interact with geographical inequalities to shape food-access outcomes.

This study aimed to determine the prevalence of food insecurity and analyze its sociodemographic and geographic determinants in Somalia. The specific objectives were to: (1) examine associations between individual-level characteristics and food insecurity among different population subgroups, and (2) assess regional disparities in food insecurity prevalence across Somalia’s diverse regions.

## Methodology

### Study design and data source

This study utilized secondary data from the 2020 Somalia Demographic and Health Survey (SDHS), which employed a cross-sectional analytical design. The SDHS is a nationally representative survey conducted by the Somalia National Bureau of Statistics in collaboration with the DHS Program (ICF International). Data were collected between February and September 2020 by trained interviewers using face-to-face interviews across urban, rural, and nomadic populations. The SDHS is a nationally representative survey designed to collect detailed demographic and health-related data, including variables related to household food insecurity and sociodemographic indicators. The dataset used in this analysis was collected from 52,154 individuals from various regions of Somalia, including urban, rural, and nomadic settings.

### Study population and sampling

The study population included individuals aged 13 years and above residing in Somalia at the time of the survey. The SDHS utilizes a multi-stage stratified cluster sampling technique to ensure broad geographic and population group representation. Stratification was performed based on region and residence type (urban, rural, and nomadic). All individuals who responded to the questions regarding food insecurity and sociodemographic factors were included in the final sample.

### Dependent variable

The dependent variable was food insecurity, measured using the SDHS food security module adapted from the Household Food Insecurity Access Scale (HFIAS). Participants were asked: ‘In the past 4 weeks, was there ever no food to eat of any kind in your house because of lack of resources to get food?’ Response options were ‘Yes’ (coded as 1, indicating food insecurity) or ‘No’ (coded as 0, indicating food security). Although the SDHS includes multiple severity levels of food insecurity, we created a binary variable to ensure adequate statistical power across all regional and demographic subgroups.

### Independent variables

Independent variables were grouped into four thematic categories:

1. Demographic characteristics: Age, sex, marital status.
2. Socioeconomic factors: Educational attainment and wealth quintiles (computed using principal component analysis of household assets, dwelling characteristics, and ownership of consumer goods, as per standard DHS methodology).
3. Geographic factors: Type of residence and region of residence across all 18 Somali administrative regions.
4. Food security indicators: The variable ‘sleep hungry’ assessed by the question: ‘In the past 4 weeks, did you or any household member go to sleep at night hungry because there was not enough food?‘”.

### Data analysis

**Fig. 1:**
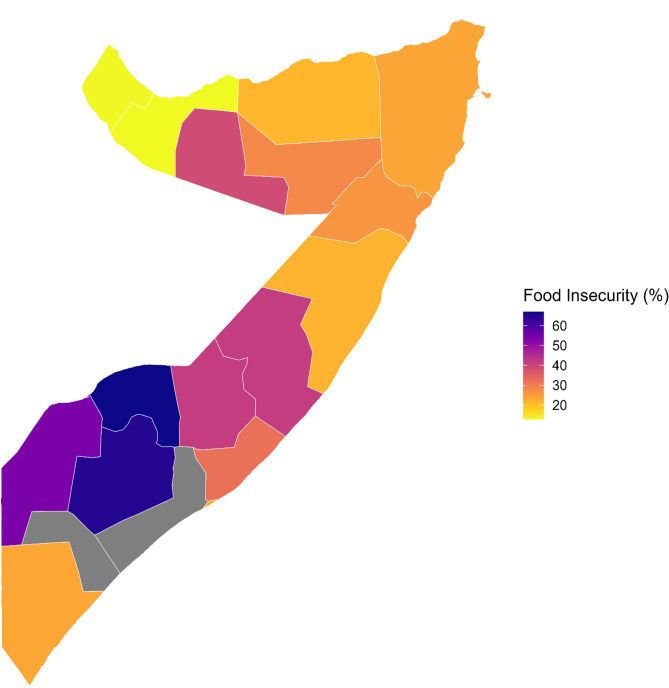
Food insecurity across Somali regions

Data analysis followed the Strengthening the Reporting of Observational Studies in Epidemiology (STROBE) guidelines, which provide a checklist of essential items for reporting cross-sectional studies, ensuring comprehensive coverage of study design, data collection, analysis methods, and result interpretation. for observational studies conducted in Stata 18.0 and R 4.3.1. After data cleaning and multiple imputations for < 5% missing values, descriptive statistics were used to characterize the sample (Table 1). Bivariate logistic regression identified crude associations between predictors and food insecurity, and odds ratios (ORs) and 95% confidence intervals (CIs) were calculated (Table 2). Variables achieving *p* < 0.25 in bivariate analysis were entered into the multivariate model, variables with *p* < 0.25 in bivariate analysis were considered for inclusion. Confounders were selected based on literature review and included age, sex, education, wealth quintile, and geographic factors. The final adjusted model (Table 2) reported AORs after controlling for age, sex, wealth, and regional clustering, with variance inflation factors of < 5, confirming no multicollinearity. Geographical mapping was performed in R Studio version 4.5.0 using choropleth visualization to depict regional disparities (Fig. 1). Polygon boundaries were obtained from the GADM database (version 2021) for Somalia and combined with food insecurity data to produce the thematic map.

## Results

Table 1 presents the socioeconomic and demographic characteristics of the 52,154 study participants. The majority of participants were aged 20–29 years (22.8%), followed by those aged 30–39 years (19.4%). Most participants had no formal education (58.3%), while 23.1% completed primary education. Regarding marital status, 67.2% were married, 18.9% never married. Rural residents comprised 48.2% of the sample, urban residents 32.1%, and nomadic populations 19.7%. Food insecurity affected 45.7% (95% CI: 44.2–47.2%) of participants, while 31.2% reported sleeping hungry in the past four weeks.

**Table 1:** Socioeconomic and demographic characteristics of study participants. Somalia demographic and health survey, 2020 (*N* = 52,154)

Variable	Category	Frequency	Percent
**Age**	13–19	426	0.82
	20–29	5,510	10.56
	30–39	10,864	20.83
	40–49	11,468	21.99
	50–59	11,573	22.19
	60+	12,313	23.61
**Education**	No education	36,992	70.93
	primary	8,935	17.13
	secondary	4,341	8.32
	Higher education	1,886	3.62
**Marital status**	Married	25,765	49.40
	Divorced	1,880	3.60
	Abandoned	399	0.77
	Widowed	2,369	4.54
	Never Married	21,741	41.69
**Wealth quintile**	lowest	17,385	33.33
	second	8,388	16.08
	middle	9,167	17.58
	fourth	8,954	17.17
	highest	8,260	15.84
**Type of residence**	Urban	24,451	46.88
	Rural	13,781	26.42
	Nomadic	13,922	26.69
**Sex**	Male	36,040	69.10
	Female	16,114	30.90
**Region of residence**	Awdal	3,055	5.86
	Woqooyi Galbeed	4,459	8.55
	Togdheer	4,374	8.39
	Sool	4,211	8.07
	Sanaag	4,571	8.76
	Bari	2,797	5.36
	Nugaal	2,956	5.67
	Mudug	3,016	5.78
	Galgaduud	2,547	4.88
	Hiraan	2,712	5.20
	MiddleShabelle	2,286	4.38
	Banadir	6,977	13.38
	Bay	932	1.79
	Bakool	2,091	4.01
	Gedo	2,613	5.01
	Lower jubba	2,557	4.90

**Fig. 2:**
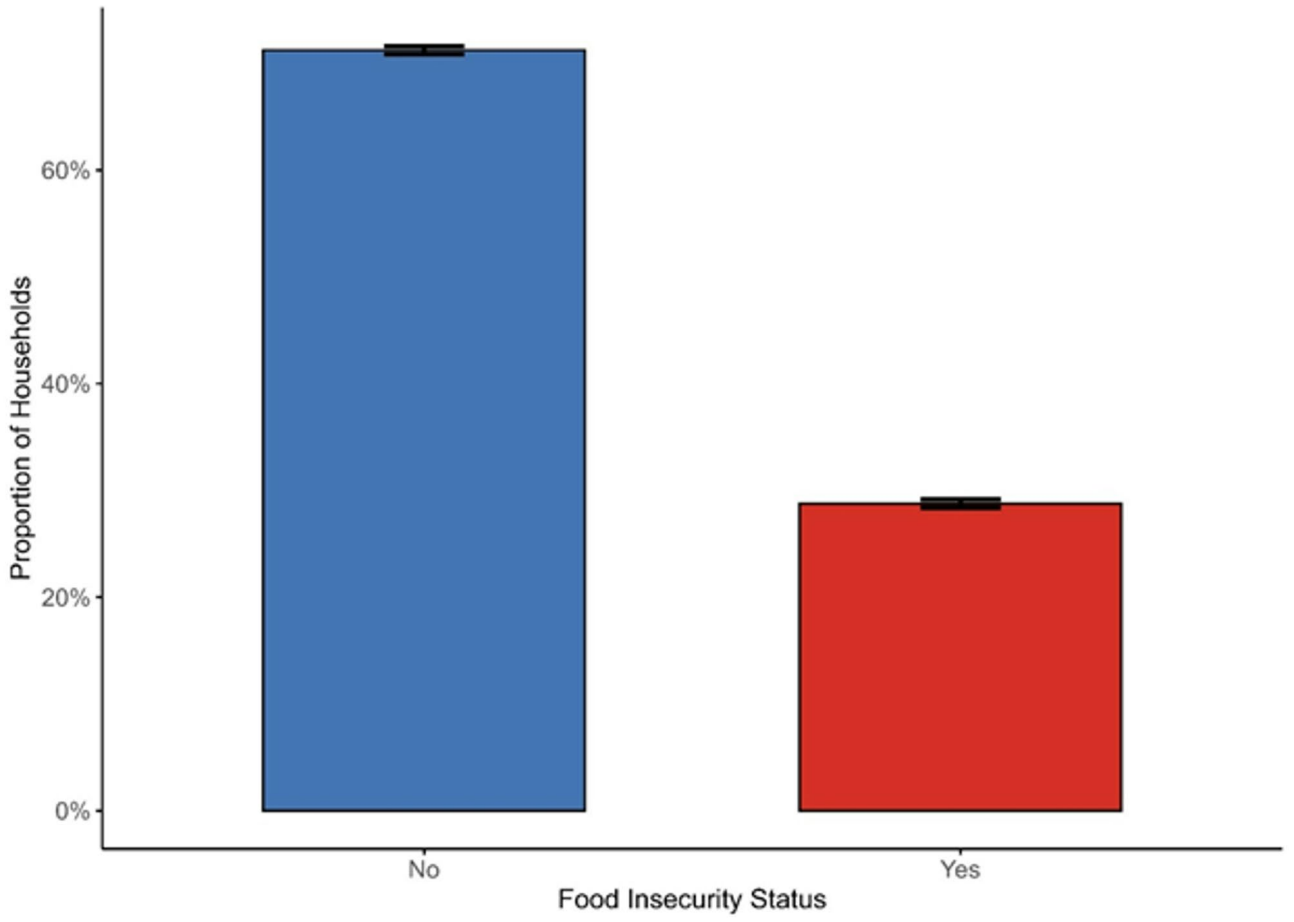
Proportion of Households Experiencing Food Insecurity in Somalia

Figure 2 shows the overall prevalence of food insecurity in Somalia, with 45.7% of participants reporting food insecurity in the past four weeks.

Table 2 presents the results of bivariate logistic regression analyses examining the associations between key socio-demographic variables and food insecurity (“food insecurity”) among 52,154 participants in Somalia. The analysis revealed significant disparities across age cohorts, with adults aged 30–39 years (OR = 1.39, *p* = 0.004), 50–59 years (OR = 1.28, *p* = 0.029), and those aged over 60 years (OR = 1.28, *p* = 0.030) demonstrating elevated odds of food insecurity compared to adolescents aged 13–19 years. Educational attainment exhibited a strong protective gradient, with primary education (OR = 0.70, *p* < 0.001), secondary education (OR = 0.36, *p* < 0.001), and higher education (OR = 0.18, *p* < 0.001) progressively reducing odds relative to no formal education. Marital status analysis identified heightened vulnerability among divorced (OR = 1.12, *p* = 0.028), abandoned (OR = 1.27, *p* = 0.023), and widowed individuals (OR = 1.16, *p* = 0.001) compared to married participants, while never-married individuals showed a marginally lower risk (OR = 0.92, *p* < 0.001). The “sleep hungry” variable demonstrates extreme associations, with those not experiencing acute hunger showing near-complete protection (OR = 0.003, *p* < 0.001). Wealth quintile analysis revealed stark socioeconomic gradients, as the second quintile (OR = 1.44, *p* < 0.001) showed an increased risk, while the highest quintile exhibited strong protection (OR = 0.11, *p* < 0.001). Female-headed households face modestly higher odds (OR = 1.09, *p* < 0.001) than male-headed households. Residence-type disparities emerged, with rural (OR = 1.34, *p* < 0.001) and nomadic populations (OR = 1.53, *p* < 0.001) experiencing greater vulnerability than urban dwellers. Regional analysis revealed extreme geographical heterogeneity, with Bakool (OR = 13.60, *p* < 0.001) and Gedo (OR = 12.46, *p* < 0.001) showing catastrophic risks relative to Awdal, while Banadir (OR = 1.95, *p* < 0.001) and Hiraan (OR = 7.82, *p* < 0.001) demonstrated intermediate vulnerability. Comprehensive bivariate analysis identifies critical population subgroups and geographic patterns requiring targeted intervention while providing foundational evidence for subsequent multivariate modeling to disentangle confounding effects.

**Table 2:** Bivariate and multivariate logistic regression analysis of factors associated with food insecurity in somalia. somalia.Demographic and health survey, 2020 (*N* = 52,154)

Variable	Category	Crude OR (95% CI)	*P*-value (Crude)	Adjusted OR (95% CI)	*P*-value (Adjusted)
**Age group**	13–19 (ref)	-	-	-	-
	20–29	1.09 [0.87, 1.37]	0.443	1.92 [1.23, 3.01]	0.004*
	30–39	1.39 [1.11, 1.74]	0.004*	2.55 [1.64, 3.96]	0.000*
	40–49	1.24 [0.99, 1.55]	0.059	2.38 [1.53, 3.70]	0.000*
	50–59	1.28 [1.03, 1.60]	0.029*	2.67 [1.71, 4.14]	0.000*
	60+	1.28 [1.02, 1.60]	0.030*	2.56 [1.65, 3.97]	0.000*
**Education level**	No education (ref)	-	-	-	-
	Primary	0.70 [0.67, 0.74]	0.000*	0.99 [0.87, 1.11]	0.821
	Secondary	0.36 [0.33, 0.39]	0.000*	0.92 [0.77, 1.10]	0.358
	Higher education	0.18 [0.15, 0.21]	0.000*	0.80 [0.59, 1.09]	0.153
**Marital status**	Married (ref)	-	-	-	-
	Divorced	1.12 [1.01, 1.24]	0.028*	1.03 [0.83, 1.28]	0.811
	Abandoned	1.27 [1.03, 1.56]	0.023*	1.25 [0.80, 1.94]	0.33
	Widowed	1.16 [1.06, 1.27]	0.001*	0.95 [0.78, 1.17]	0.628
	Never married	0.92 [0.89, 0.96]	0.000*	1.02 [0.92, 1.12]	0.749
**Sleep hungry**	Yes (ref)	-	-	-	-
	No	0.003 [0.003, 0.004]	0.000*	0.004 [0.0036, 0.0042]	0.000*
**Wealth quintile**	Lowest (ref)	-	-	-	-
	Second	1.44 [1.37, 1.52]	0.000*	0.75 [0.65, 0.86]	0.000*
	Middle	0.77 [0.73, 0.81]	0.000*	0.65 [0.57, 0.76]	0.000*
	Fourth	0.36 [0.34, 0.39]	0.000*	0.43 [0.36, 0.50]	0.000*
	Highest	0.11 [0.10, 0.12]	0.000*	0.22 [0.18, 0.27]	0.000*
**Sex of household head**	Male (ref)	-	-	-	-
	Female	1.09 [1.05, 1.13]	0.000*	1.18 [1.07, 1.29]	0.001*
**Type of residence**	Urban (ref)	-	-	-	-
	Rural	1.34 [1.28, 1.40]	0.000*	0.83 [0.74, 0.93]	0.002*
	Nomadic	1.53 [1.47, 1.60]	0.000*	0.77 [0.66, 0.89]	0.001*
**Region**	Awdal (ref)	-	-	-	-
	Woqooyi Galbeed	0.98 [0.86, 1.13]	0.797	0.94 [0.74, 1.20]	0.608
	Togdheer	1.87 [1.64, 2.12]	0.000*	0.82 [0.64, 1.03]	0.088
	Sool	2.38 [2.10, 2.70]	0.000*	1.01 [0.80, 1.27]	0.963
	Sanaag	2.10 [1.85, 2.38]	0.000*	0.94 [0.75, 1.18]	0.603
	Bari	4.26 [3.74, 4.85]	0.000*	0.79 [0.62, 1.01]	0.061
	Nugaal	1.80 [1.57, 2.07]	0.000*	1.02 [0.79, 1.31]	0.889
	Mudug	2.64 [2.32, 3.02]	0.000*	1.40 [1.09, 1.79]	0.008*
	Galgaduud	4.78 [4.19, 5.45]	0.000*	2.06 [1.61, 2.65]	0.000*
	Hiraan	7.82 [6.87, 8.90]	0.000*	2.56 [2.01, 3.26]	0.000*
	Middle Shabelle	3.19 [2.78, 3.66]	0.000*	1.50 [1.16, 1.94]	0.002*
	Banadir	1.95 [1.73, 2.20]	0.000*	1.19 [0.94, 1.50]	0.129
	Bay	4.81 [4.07, 5.69]	0.000*	1.31 [0.92, 1.86]	0.13
	Bakool	13.60 [11.83, 15.64]	0.000*	2.32 [1.78, 3.03]	0.000*
	Gedo	12.46 [10.91, 14.23]	0.000*	2.41 [1.87, 3.11]	0.000*
	Lower Juba	2.07 [1.80, 2.38]	0.000*	0.61 [0.47, 0.79]	0.000*

Table 2 presents both crude and adjusted associations between sociodemographic factors and food insecurity among 52,154 participants in Somalia. The bivariate analysis revealed significant age-related disparities, with adults aged 30–39 years (crude OR = 1.39, *p* = 0.004), 50–59 years (crude OR = 1.28, *p* = 0.029), and those over 60 years (crude OR = 1.28, *p* = 0.030) demonstrating elevated odds compared to adolescents aged 13–19 years. After multivariate adjustment, age effects became more pronounced, with all adult age groups showing significantly higher adjusted odds ratios ranging from 1.92 to 2.67 (all *p* < 0.05).

Educational attainment exhibited a strong protective gradient in crude analysis, with higher education reducing odds by 82% (crude OR = 0.18, *p* < 0.001) compared to no formal education. However, after adjustment for confounders, educational effects were attenuated and became non-significant, suggesting that education’s protective effect may be mediated through other socioeconomic factors such as wealth and residence type. Marital status showed differential crude effects, with divorced (crude OR = 1.12, *p* = 0.028), abandoned (crude OR = 1.27, *p* = 0.023), and widowed individuals (crude OR = 1.16, *p* = 0.001) at higher risk than married participants. These associations disappeared after multivariate adjustment, indicating confounding by other sociodemographic factors. The “sleep hungry” variable demonstrated the strongest association in both crude and adjusted models, with those not experiencing acute hunger showing dramatically reduced odds (crude OR = 0.003; adjusted OR = 0.004, both *p* < 0.001). Wealth quintile analysis revealed stark gradients that persisted after adjustment, with the highest quintile conferring strong protection in both crude (OR = 0.11) and adjusted (OR = 0.22) models (both *p* < 0.001). Female-headed households maintained elevated risk in both crude (OR = 1.09) and adjusted (OR = 1.18) analyses.

Regional disparities remained pronounced after adjustment, with Bakool (crude OR = 13.60; adjusted OR = 2.32) and Gedo (crude OR = 12.46; adjusted OR = 2.41) exhibiting the highest risks compared to Awdal reference region (all *p* < 0.001). Interestingly, rural and nomadic residence types showed protective effects in adjusted models (rural adjusted OR = 0.83, nomadic adjusted OR = 0.77) compared to their crude associations, suggesting that regional factors may mediate residence-type effects.

## Discussion

This nationally representative analysis of 52,154 Somali participants revealed several key patterns in food insecurity distribution. Nearly half (45.7%) of participants experienced food insecurity, with stark educational gradients showing higher education, reducing odds by 82% compared to no formal education. Older adults, divorced and widowed individuals, and female-headed households demonstrated elevated vulnerability. The high vulnerability observed among older adults in Somalia contrasts with patterns in countries with established social protection systems. In Somalia’s context, the collapse of formal social security systems since 1991, combined with displacement and clan-based conflicts, has left older adults particularly exposed. The protective effect observed for never-married individuals likely reflects the absence of dependent family members and greater mobility in seeking livelihood opportunities, consistent with Somalia’s pastoral traditions. This comprehensive cross-sectional analysis sought to examine the complex interplay of sociodemographic and geographic determinants shaping household food insecurity across Somalia’s diverse population subgroups and regions using nationally representative data from 52,154 participants. This investigation aimed to address critical knowledge gaps in understanding how individual-level characteristics intersect with geographic disparities to influence food access outcomes in one of the world’s most food-insecure nations. Food insecurity remains a pervasive challenge across sub-Saharan Africa, and studies have consistently demonstrated that over 30% of the regional population experiences severe food access limitations, making it the global epicenter of hunger-related vulnerabilities [23]. Research from neighboring contexts has revealed that socio-demographic factors operate through complex pathways to determine household food security status, with educational attainment, marital transition, and wealth status emerging as influential determinants [24–26]. The urgency of this investigation is underscored by evidence that food insecurity extends far beyond immediate nutritional deficits and serves as a fundamental driver of poor health outcomes, reduced cognitive development, and increased mortality rates across vulnerable populations [27, 28]. Contemporary research has demonstrated that approximately 828 million people globally experience chronic hunger, with Sub-Saharan Africa bearing a disproportionate burden that threatens to undermine progress toward achieving Sustainable Development Goals [23, 29].

The age-related patterns observed in food insecurity demonstrate complex vulnerability profiles that vary significantly across different life stages, with the existing literature revealing both consistent and contrasting findings regarding age-specific risks. Research from the United States indicates that midlife adults experience the highest vulnerability, with late midlife (37.5%) and early midlife (36.0%) populations showing elevated food insecurity rates compared with younger adults (33.7%) and older adults (20.2%) [30]. This midlife vulnerability pattern contrasts with findings from other contexts, where older adults demonstrate increased susceptibility, as evidenced by studies showing that adults aged 70–74 years have significantly higher odds of food insecurity (OR = 1.405) [31]. The differential age effects may be attributed to varying social protection systems, employment patterns, and lifecycle economic pressures, where midlife adults face competing demands, including supporting both dependent children and aging parents, while potentially experiencing career instability [30, 32]. Additionally, research among older adults reveals that food insecurity affects approximately 7.1% of US households with adults over 65 years old, with those living alone experiencing even higher rates at 9.5% [33]. International studies from Ghana report a food insecurity prevalence of approximately 28% among adults aged ≥ 50 years [34]. Age-related vulnerability appears to be mediated by role-related risk factors, with households containing children, disabled working-age adults, or individuals experiencing job loss representing 74.8% of food insecure households [32].

Educational attainment consistently emerges as a powerful protective factor against food insecurity across diverse geographical and cultural contexts, with research demonstrating a clear gradient effect, where higher education levels correlate with progressively lower food insecurity risks. Studies from Ethiopia reveal that households with no education face substantially higher adjusted odds of food insecurity (AOR: 5.62), with risk decreasing through primary (AOR: 2.58) and secondary education (AOR: 2.20) [35]. This protective gradient is further supported by research during the COVID-19 pandemic, in which older adults with college or graduate degrees experienced smaller increases in food insecurity (4.06%) than those with lower educational attainment [36]. The protective mechanism of education operates through multiple pathways including enhanced employment opportunities, improved financial literacy, better health knowledge, and increased access to social networks and resources [35, 37]. Rural studies from Ethiopia demonstrate extreme educational disparities, with illiterate household heads showing dramatically elevated odds ratios (AOR = 113.4) compared to those with higher education [37]. International evidence from older adult populations confirms this pattern, where lower education levels are associated with higher odds of food insecurity (OR = 3.355) [31], whereas research from Ghana indicates that education serves as a significant predictor of food security status among older adults [34]. The consistency of educational effects across different contexts suggests that investment in education represents a fundamental strategy for reducing long-term vulnerability to food insecurity at both individual and population levels [35, 36].

The differential vulnerability patterns observed across residence types align with extensive international research demonstrating consistent rural-urban food insecurity gradients across diverse geographical contexts. Studies from Togo reveal that rural respondents exhibit significantly higher relative risk ratios for severe food insecurity compared to urban populations, with rural residence independently associated with a 37% increased risk, even after controlling for socioeconomic factors [38]. This pattern extends beyond Sub-Saharan Africa, as research from the United States demonstrates similar urban-rural disparities in childhood food insecurity, where rural households consistently face higher food expenditure-to-income ratios than their urban counterparts [39, 40]. The heightened vulnerability of nomadic populations observed in Somalia reflects broader regional patterns, with studies of East and West African pastoralists confirming that remoteness and sociopolitical marginalization result in substantially higher food insecurity rates among mobile populations than among sedentary communities [41, 42]. Research examining food environments during the COVID-19 pandemic in Kenya corroborates these findings, demonstrating that rural respondents reported access to significantly more diverse food environments yet paradoxically experienced persistent food access challenges due to structural inequalities [43].

The extreme regional heterogeneity in food insecurity prevalence illustrated in Fig. 1 reflects the broader continental patterns of geographical clustering documented across Somali regions, where geographical proximity to conflict zones and climate-vulnerable areas creates distinct vulnerability hotspots. Continental-scale spatiotemporal analysis identified Somalia as the most likely significant space-time cluster for severe food insecurity across Africa, with the highest likelihood ratio and relative risk concentrated in the Horn of Africa region between 2015 and 2021 [13]. This geographical concentration aligns with broader regional assessments, indicating that the Horn of Africa constitutes one of the world’s most acute food insecurity emergencies, with over 37 million people experiencing crises or emergency levels of food insecurity [44]. The geographic heterogeneity observed across Somali regions corresponds to documented patterns of population displacement, where geographical proximity to conflict zones and climate shocks create differential vulnerability patterns that drive internal migration flows toward urban centers with greater humanitarian service accessibility [45]. Research on drought and mortality patterns in Somalia confirms that geographical disparities in food insecurity translate into differential demographic outcomes, with excess mortality concentrated in regions that experience the intersection of environmental shocks and limited institutional capacity [46]. These spatial patterns underscore the critical importance of place-based interventions that recognize the geographic clustering of vulnerability factors, rather than uniform national approaches to food security programming.

These findings suggest several targeted public health interventions. Educational initiatives should prioritize adult literacy programs and vocational training in rural and nomadic communities. For geographic disparities, mobile health units and conditional cash transfer programs should target high-risk regions like Bakool and Gedo. Vulnerable demographic groups require specific interventions: widow support programs, elderly care services, and women’s empowerment initiatives. Nomadic populations need mobile service delivery models that account for seasonal migration patterns.

### Study limitations

This analysis of food insecurity determinants in Somalia, while offering valuable insights, has several limitations. The use of self-reported data may introduce social desirability bias, leading to the potential underreporting of food deprivation. The cross-sectional nature of the study limits the ability to determine causal relationships between variables, such as education, wealth, and food insecurity. Geographical masking of survey cluster locations may have caused spatial misclassification, especially in nomadic regions, affecting the accuracy of the regional patterns. The exclusion of populations in conflict-affected areas likely introduced selection bias by omitting those most vulnerable. The binary classification of food insecurity oversimplifies the complexity of food access and fails to capture aspects, such as dietary diversity and chronicity. In addition, unmeasured factors such as clan influence or informal support systems may have confounded the observed associations.

## Conclusion

This nationally representative study revealed that food insecurity affects nearly half of Somalia’s population, with pronounced educational gradients, demographic vulnerabilities, and extreme regional disparities.

These findings highlight the need for targeted, multi-sectoral interventions that address educational infrastructure, provide support for vulnerable demographic groups, and implement place-based strategies. Future research should examine causal pathways and evaluate intervention effectiveness in Somalia’s fragile state context.

## Data Availability

This study used secondary data from the publicly accessible 2020 Somalia Demographic and Health Survey (SDHS). No new data were collected.
